# COVID-19 infections in infants

**DOI:** 10.1038/s41598-022-11068-0

**Published:** 2022-05-11

**Authors:** Małgorzata Sobolewska-Pilarczyk, Maria Pokorska-Śpiewak, Anna Stachowiak, Magdalena Marczyńska, Ewa Talarek, Agnieszka Ołdakowska, Izabela Kucharek, Adam Sybilski, Anna Mania, Magdalena Figlerowicz, Katarzyna Mazur-Melewska, Paulina Potocka, Artur Sulik, Barbara Hasiec, Martyna Stani, Paulina Frańczak-Chmura, Barbara Szczepańska, Ilona Pałyga-Bysiecka, Przemysław Ciechanowski, Joanna Łasecka-Zadrożna, Izabela Zaleska, Leszek Szenborn, Urszula Dryja, Ernest Kuchar, Sławomira Niedźwiecka, Bolesław Kalicki, Robert Flisiak, Małgorzata Pawłowska

**Affiliations:** 1 Department of Infectious Diseases and Hepatology, Faculty of Medicine, Collegium Medicum, Nicolaus Copernicus University, Bydgoszcz, Poland; 2 Department of Childrens Infectious Diseases, Medical University of Warsaw, Warsaw, Poland; 3 Regional Hospital of Infectious Diseases, Warsaw, Poland; 4 2Nd Department of Pediatrics, Centre of Postgraduate Medical Education, Warsaw, Poland; 5 Department of Pediatrics and Neonatology with Allergology Center, Central Clinical Hospital of the Ministry of the Interior, Warsaw, Poland; 6 Department of Infectious Diseases and Child Neurology, Poznań University of Medical Sciences, Poznan, Poland; 7 Department of Pediatric Infectious Diseases, Medical University of Białystok, Białystok, Poland; 8 Department of Childrens Infectious Diseases, Provincial Jan Boży Hospital in Lublin, Lublin, Poland; 9 Collegium Medicum Jan Kochanowski University, Kielce, Poland; 10 Department of Pediatrics and Infectious Diseases, Regional Hospital in Szczecin, Szczecin, Poland; 11 Department of Pediatrics and Infectious Diseases, Wrocław Medical University, Wroclaw, Poland; 12 Department of Pediatrics Infectious Diseases, Medical University of Łódź, Łódź, Poland; 13 Department of Pediatrics With Clinical Assessment Unit, Medical University of Warsaw, Warsaw, Poland; 14 Department of Pediatrics Infectious Diseases, Pomeranian Center of Infectious Diseases and Tuberculosis in Gdańsk, Gdansk, Poland; 15 Department of Pediatrics, Pediatrics Nephrology and Allergology, Military Institute of Medicine, Warsaw, Poland; 16 Department of Infectious Diseases and Hepatology, Medical University of Białystok, Bialystok, Poland

**Keywords:** Infectious diseases, SARS-CoV-2, Respiratory signs and symptoms

## Abstract

The study aimed to analyse the clinical course of COVID-19 in 300 infants, selected from 1283 children diagnosed with COVID-19 between March and December 2020, registered in the SARSTerPED multicenter database. Most of the infants were registered in October and November 2020. 44% of the group were girls, and 56% were boys. At diagnosis, the most common symptoms were fever in 77% of the children, cough in 40%, catarrh in 37%. Pneumonia associated with COVID-19 was diagnosed in 23% of the children, and gastrointestinal symptoms in 31.3%. In 52% of the infants, elevated levels of D-dimers were observed, and in 40%, elevated levels of IL-6 serum concentration were observed. During the second wave of the pandemic, 6 times more infants were hospitalized, and the children were statistically significantly younger compared to the patients during the first wave (3 months vs 8 months, *p* < 0.0001 respectively). During the second wave, the infants were hospitalized for longer. COVID-19 in infants usually manifests as a mild gastrointestinal or respiratory infection, but pneumonia is also observed with falls in oxygen saturation, requiring oxygen therapy. Gastrointestinal symptoms are common in infants infected with SARS-CoV-2, and infant appetite disorders may lead to hospitalization. The clinical course of the disease differed significantly between the first and second wave of the pandemic. It seems that infants may play a role in the transmission of SARS-COV-2 infections in households, despite mild or asymptomatic courses; eating disorders in infants should be an indication for COVID-19 testing.

## Introduction

In Poland, the first case of COVID-19 was registered on March 04, 2020, and the first infected child in the SARSTer-PED study was registered on March 16, 2020. In the study by Raciborski et al., concerning 12,877 people infected with SARS-CoV-2 in the first two months of the pandemic, the infection rate for the entire population was 33.2 cases per 100,000 inhabitants. The lowest infection rate was recorded in the youngest age groups, 0–4 and 5–9 years, amounting to 6.5 and 8 per 100,000 inhabitants, respectively^1^.

The clinical picture of COVID-19 is diverse. The course of the disease may be asymptomatic, mild symptomatic, without pneumonia or with mild pneumonia, and symptoms resolve within 1–2 weeks. About 15% of infected cases develop a severe form of COVID-19 with pneumonia, accompanied by dyspnoea and hypoxia, and 5% of symptomatic patients develop thrombosis, septic shock, and multi-organ failure due to a cytokine storm resulting from an abnormal over-response of the immune system. The most frequently reported clinical symptoms of SARS-CoV-2 infection are fever, cough, dyspnoea, sore throat, muscle pain, headache, catarrh, and changes in the senses of smell and taste^2–6^.

COVID-19 infection in children has similar symptoms as in adult patients, but more often in the pediatric population, the disease resembles a mild cold^7^. In an analysis by Kainth et al.^8^, fever was present in 86% of children, lower respiratory symptoms in 60%, and gastrointestinal symptoms in 62%. In the observations of American authors, most of the sick infants had mild infections^5,9^.

The reasons for the milder course of infection among children are ambiguous. Hypotheses about possible cross-resistance due to the presence of antibodies produced after infections with other hCoVs in early childhood have been raised, as demonstrated in large population studies conducted in Norway and China^10,11^. Co-infections with another hCoV, influenza virus or RSV were found in 47.2% of children^10^. The course of infection in children is also influenced by a lower incidence of comorbidities and differences in immune responses, which reduce the risk of a cytokine storm^12^. It has also been demonstrated that the expression of the angiotensin converting enzyme type 2 (ACE2) receptor, to which SARS-CoV-2 binds to access cells, is lowest in the nasal epithelium of young children and increases with age^13,14^.

The aim of the study was to evaluate the clinical course of COVID-19 in infants based on the analysis of the pediatric element of the SARSTer multicentre database (SARSTer-PED).

## Methods

The study included 300 Caucasian infants aged 0–12 months with a documented positive result in a SARS-CoV-2 nucleic acid amplification test who reported to the emergency room of hospitals in 13 Polish centers. Of the 300 infected children, 94% were hospitalized. Infants were enrolled in the study after obtaining the informed consent of the parent and/or legal guardian.

The analyzed group was selected from among 1,283 children up to 18 years of age, who were diagnosed and treated in the period from March 1, 2020 to December 31, 2020.

For the purpose of this study, two waves of the pandemic were defined: the first wave from March to August 2020, and the second wave from September to December 2020. The courses of COVID-19 in children diagnosed during the first and second waves of the pandemic were compared.

Epidemiological interviews were conducted based on electronic questionnaires. The analysis considered data on demographics, clinical signs, laboratory abnormalities, and comorbidities.

Demographic data included age and sex. Epidemiologic data included known exposure to a person with confirmed SARS-CoV-2 infection (in the household or otherwise), history of any international travel during 14 days before the disease onset, duration of symptoms before presentation, and comorbidity. All symptoms at the time of admission and during hospitalization (if applicable) were documented. Patients´ COVID-19 illness severity was classified as mild, with no requirement for supplemental oxygen, moderate, with supplemental oxygen noninvasive respiratory support, or severe with a requirement for mechanical ventilation.

The indication for hospitalization was fever, anorexia, or symptoms of infections in the respiratory tract such as a cough, or in the digestive system (diarrhea, vomiting).

High fever was defined as body surface temperature of > 38.5 °C, and low-grade fever was defined as 37.0 °C–38.5 °C.

Laboratory testing and imaging results (if performed due to the clinical indications) were recorded. Laboratory confirmation of SARS-CoV-2 infection was diagnosed by a positive result of real-time polymerase chain reaction (RT-PCR) on a nasopharyngeal swab, performed in certified molecular diagnostics laboratories using certified RT-PCR testing methods for the SARS-CoV-2 infection. The diagnosis of COVID-19 was based on the International Statistical Classification of Diseases and Health Problems (ICD-10) diagnostic code U07.1.

Statistical analysis was performed using MedCalc Statistical Software version 19.2.1 (MedCalc, Ostend, Belgium, https://www.medcalc.org). Categorical variables were compared using the chi-square test. Continuous variables were presented as medians with interquartile ranges (IQRs) and were compared using the Mann–Whitney test. A two-sided *p* value of < 0.05 was considered significant.

### Ethical statement

The study was performed in accordance with the ethical standards in the 1964 Declaration of Helsinki and its later amendments. The local Ethics Committee of the Regional Medical Chamber in Warsaw approved this study (No KB/1270/20; date of approval: 3 April 2020).

## Results

Children under 1 year of age diagnosed with COVID-19 accounted for 23.4% (300/1283) of the studied pediatric population.

During the first wave of the pandemic (March to August 2020), 10.5% (49/465) of the diagnosed children were infants, and during the second wave (September to December, 2020), 30.7% (251/818) were infants.

Of the 300 infants diagnosed with COVID-19, boys predominated and constituted 56%, while 44% were girls. The age of the children at the time of diagnosis ranged from 5 days to 12 months, and the median age was 4 months (Table 1). Infants under 1 month of age constituted 6% (18/300) of the study group.

**Table 1:** Baseline characteristics of the study group (*N* = 300).

Feature		Number
Age	Range	5 days–12 months
	Median (IQR) (months)	4 (2; 8)
Sex	Male/female	169 (56)/131 (44)
Household contact with an infected family member	Yes	177 (59)
Confirmed other contact	Yes	2 (1)
Confirmed COVID-19 in a family member (data available for 173 patients)	Before the diagnosis established in the child	54 (31)
	Diagnosis in the child established simultaneously	99 (57)
	After the diagnosis established in the child	20 (12)
Duration to negative PCR testing for SARS-CoV-2 infection (data available for 28 patients)	Days, median (IQR)	18 (11; 21)
Hospitalization	Yes	282 (94)
	Lasting one-day	46 (15)
	Lasting over 24 h	236 (79)
	Duration (days), Median (IQR)	5 (3; 8)
Duration of infection before admission	Days Median (IQR)	2 (1; 3)
International travel	(During 14 days before the onset of the disease)	6 (2)
Comorbidities including	Present	35 (12)
	Congenital disabilities	7
	Epilepsy	4
	Prematurity	3
	Atopic dermatitis	3
	Bronchopulmonary dysplasia	3
	Immunodeficiency	1
Wave of COVID-19 pandemic	(1st/2nd)	49 (16)/251 (84)

Data are presented as Number (%), unless otherwise indicated.

**Figure 1:**
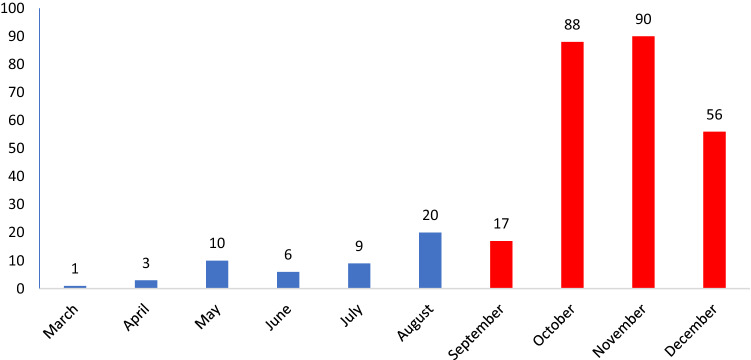
The number of reported COVID-19 cases in infants diagnosed in the subsequent months of 2020, divided into two waves of the pandemic.

A significant increase in the number of cases among the youngest children was registered in October, November, and December 2020 (2nd wave of the pandemic). (Fig. 1).

In 59% of the cases, contact with an infected family member in the household was confirmed. Based on the interviews, it was found that 2% of the children had been traveling abroad before the onset of symptoms of COVID -19.

282 infants were hospitalized, while a further 18 were checked in hospital and sent home due to no indication for hospitalization.

The duration of inpatient treatment in 79% of the patients was on average 5 days (median 3 to 8 days), and in 15% of the patients, the length of the hospital stay was 1 day (Table 1).

**Figure 2:**
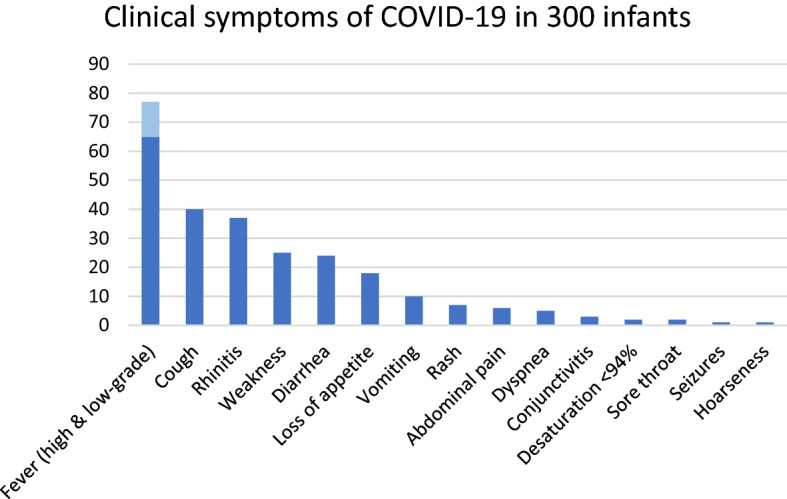
Clinical symptoms of COVID-19 in 300 infants. Data are presented as prevalence of the symptoms [%].

At the diagnosis, the most common symptom was high fever or low-grade fever, which was reported in 196 (65%) and in 35 (12%) children, respectively. Further symptoms included a cough in 119 (40%) children and catarrh in 101 (37%). Other reported clinical signs of COVID-19 in children were weakness in 75 children (25%), diarrhea in 73 (24%), loss of appetite in 55 (18%), vomiting in 29 (10%), rash in 21 (7%), abdominal pain in 18 (6%), and dyspnoea in 16 (5%) (Fig. 2).

Severity of disease was classified as mild 276 (97.9%), moderate 6 (2%), and severe 0. An asymptomatic course was recorded in 32 (11%) patients.

Comorbidities were reported in 12% of infants, including congenital disabilities, epilepsy, prematurity, atopic dermatitis, bronchopulmonary dysplasia, and immunodeficiency (Table 1).

In the analyzed laboratory test deviations, 52% (74/141) of the patients had an elevated concentration of D-dimers > 500 ng/mL, and 40% (32/80) of the cases had an elevated concentration of Interleukin-6 (IL-6) > 7 pg/mL.

Antipyretic drugs were used in 115 (38%) patients, including paracetamol in 62 (54%), ibuprofen in 12 (10%), and a combination of paracetamol and ibuprofen in 41 (36%). No infants received remdesivir, tocilizumab, or anticoagulants.

Among the additional diseases found in infants with COVID-19 during the first and second waves, urinary tract infections (22/300), viral gastrointestinal infections (11/300), and otitis media (9/300) were the most frequently diagnosed.

183 of the 282 hospitalized children (65%) had chest radiographs; 70 (25%) had abnormal findings on the radiograph. Pulmonary lesions in CXR included patchy consolidation with lower lung distribution, peribronchial thickening, and peribronchial consolidation. Computed tomography was not performed for any infant.

Based on clinical symptoms and chest X-ray examination, pneumonia was diagnosed in the course of SARS-CoV-2 infection in 70 (23.3%) children. Gastrointestinal symptoms were observed in 19 (27%) of them, and 6 children with saturation < 94% required oxygen therapy. One child was treated in an intensive care unit but did not require mechanical ventilation. Boys suffered from pneumonia more often (67% vs. 33%). In the infants with pneumonia, fever above 38.5 °C was observed in 70%, a cough in 46%, and in 27% there were also gastrointestinal symptoms. In this group of infants, comorbidities occurred in 16% of the patients. Laboratory abnormalities in infants with pneumonia included elevated IL-6 levels to an average of 9.1 pg/ml, and elevated D-dimer levels to an average of 608 ng/ml. There were no statistically significant differences in C-reactive protein (CRP) concentrations, leukocytes, IL-6, D-dimers and aminotransaminases in infants with pneumonia compared to children with COVID-19 without pneumonia.

Gastrointestinal symptoms during COVID-19 occurred in 94 (31.3%) children: 53 boys and 41 girls. In 69% of the patients, gastrointestinal symptoms were accompanied by a fever above 38.5 °C. In this group of children, 11% had comorbidities. Among infants with gastrointestinal symptoms, elevated levels of IL-6 and D-dimers were recorded, compared to children without gastrointestinal symptoms, but the differences were not statistically significant. In this group of children, there were no increases in concentrations of CRP, leukocytes or aminotransaminases. No statistically significant differences were observed in CRP levels, leukocytes and alanine aminotransferase (ALT) activity compared to the group of children without gastrointestinal symptoms.

The courses of COVID-19 in children diagnosed during the first and second waves of the pandemic were compared. During the second wave of the pandemic, 6 times more children under 1 year of age were hospitalized (242 vs 40, *p* = 0.0001). In addition, children were statistically significantly younger compared to the patients during the first wave (*p* < 0.0001). Children hospitalized during the second wave had significantly less frequent household contacts with infected family members (53% vs 90%). During the second wave, infants were also hospitalized for significantly longer (*p* < 0.0001), (Table 2).

**Table 2:** Baseline epidemiological characteristics of infants with COVID-19 during the 1st and 2nd waves of the pandemic.

Characteristics	Patients diagnosed between March and August 2020 *N* = 49	Patients diagnosed between September and December 2020 *N* = 251	*P*
Age (months) median (IQR)	8 (4; 10)	3 (1; 7)	** < 0.0001**
Sex (male/female)	24 (49)/ 25 (51)	145 (58)/ 106 (42)	0.25
Hospitalized	40 (82)	242 (96)	**0.0001**
Duration of hospitalization (days) median (IQR)	1 (1; 5)	4 (2; 7)	** < 0.0001**
Household contact with an infected family member	44 (90)	133 (53)	** < 0.0001**
Confirmed other contact	0	2 (1)	0.53
International travel during 14 days before the onset of symptoms	2 (4)	4 (2)	0.25
Comorbidities	4 (8)	31 (12)	0.40

Data are presented as Median (IQR) or *n* (%), as appropriate. Significance values are in Bold.

Children hospitalized in the second wave of the pandemic more often developed fever (*p* = 0.008), weakness (*p* = 0.02), and decreased appetite (*p* = 0.01), (Table 3). When analyzing the results of studies in particular periods of the epidemic, statistically significant higher procalcitonin (PCT) concentration (*p* = 0.002), and decreased concentrations of Hemoglobin (Hgb) and Red blood cells (RBC) *p* = 0.01 vs *p* = 0.02, were observed more often during the second wave. No statistically significant differences were observed in the concentrations of CRP, IL-6, D-Dimers, and aminotransferases in infected infants during the first and second pandemic waves (Table 4).

**Table 3:** Clinical presentation of infants with COVID-19 during the 1st and 2nd waves of the pandemic.

Clinical presentation/symptoms	Patients diagnosed between March and August 2020 *N* = 49	Patients diagnosed between September and December 2020 *N* = 251	*P*
Asymptomatic course of disease	9 (18)	23 (9)	0.05
Pneumonia related to COVID-19	8 (16)	62 (25)	0.20
Gastrointestinal symptoms	10 (20)	84 (33)	0.07
Fever	24 (49)	172 (69)	**0.008**
Cough	19 (39)	100 (40)	0.88
Rhinitis	13 (27)	88 (35)	0.53
Weakness	6 (12)	69 (27)	**0.02**
Diarrhea	7 (14)	66 926)	0.07
Sore throat	2 (4)	3 (1)	0.14
Vomiting	4 (8)	25 (10)	0.69
Abdominal pain	1 (2)	17 (7)	0.20
Loss of appetite	3 (6)	52 (21)	**0.01**
Dyspnea	2 (4)	14 (6)	0.67
Rash	3 (6)	18 (7)	0.79
Conjunctivitis	3 (6)	7 (3)	0.23
Seizures	0	4 (2)	0.37

Data are presented as numbers (%). Significance values are in Bold.

**Table 4:** Diagnostics and treatment of infants with COVID-19 during the 1st and 2nd waves of the pandemic.

Characteristics	Patients diagnosed between March and August 2020 *N* = 49	Patients diagnosed between September and December 2020 *N* = 251	*P*
**Laboratory testing**			
Red blood cells (T/L)	4.5 (4.2; 4;8)	4.2 (3.7; 4.7)	**0.02**
Hemoglobin (g/dL)	12.3 (11.3; 12.9)	11.3 (10.7; 12.5)	**0.01**
Leukocyte count (10^3^/µL)	9.8 (7.4; 12.8)	11 (7.9; 14.7)	0.29
CRP (mg/L)	5.0 (2.0; 5.0)	3.69 (0.7; 6.9)	0.54
PCT (ng/mL)	0.05 (0.05; 0.08)	0.09 (0.05; 0.18)	**0.002**
Interleukin-6 (pg/mL)	3.4 (2.3; 8.0)	7.8 (3.4; 20.0)	0.10
D-Dimer (ng/mL)	425 (332; 1103)	606 (367; 1050)	0.54
ALT (IU/L)	24.5 (21.0; 37.5)	29.0 (20.7; 38.0)	0.83
AST (IU/L)	51.0 (40.0; 62.5)	48.0 (38.0; 58.7)	0.51
**Diagnosis and treatment**			
Diagnoses additional to COVID-19	5 (10)	79 (31)	**0.002**
Azithromycine	15 (30)	34 (14)	**0.003**
Empirical antibiotic	3 (6)	84 (33)	**0.0001**

Data are presented as Median (IQR) or *n* (%), as appropriate.

ALT—alanine aminotransferase; AST—aspartate aminotransferase; CRP—C-reactive protein; PCT—procalcytonin. Significance values are in Bold.

During the second wave, additional diagnoses of COVID-19 were significantly more frequent (*p* = 0.002), and empirical antibiotic therapy was used more often (*p* = 0.0001) (Table 4).

## Discussion

The incidence of COVID-19 among children in various countries is estimated at 1–2% of all reported cases, and in countries with a higher number of tests performed, rates were as high as 5–13%^5,15–20^. In the study by Dong et al.^21^, concerning 2143 children with COVID-19, most infections were mild (50.9%) or moderate (38.8%), and in 4.4% of children the course of the disease was asymptomatic. Similarly, in the study by Leibowitz et al.^9^, the majority of febrile COVID-19 infants had a mild course of the infection, and only 2 out of 20 children required supplemental oxygen.

In the SARSTer-PED study, 97.9% of the infants had a mild course of the disease. However, it was found that infants were more prone to a severe course of the disease than older children, and accounted for the highest percentage (32%) of seriously ill children^21^. However, in our study, no infant had a severe course of the disease. Similar results were published by Kainth et al.^8^.

It should be assumed that the actual incidence of COVID-19 in children is underestimated, due to the lack of diagnoses of mild and asymptomatic courses. Reports of asymptomatic infections in children and infants were published by researchers at an early stage of the pandemic. Asymptomatic infections were described and diagnosed within a study on family foci of the disease^22–26^. Similar observations were also made in individual patients under 1 month of age who were diagnosed during hospitalization related to vertical infections. In our study, the asymptomatic course of COVID-19 concerned 11% of the infected infants. Therefore, it is important to test all household contacts and plan SARS-CoV-2 seroprevalence tests in the pediatric population, in order to estimate the actual number of infections. At the same time, it is justified to diagnose infants and young children to limit the transmission of SARS-CoV-2, especially in the context of low-symptomatic infections.

In our study, boys (56%) predominated among the infected children up to 1 year of age, although no statistically significant difference in the number of patients was found between boys and girls. A similar proportion of infected boys were found in studies of Chinese (56.6%) and American (57%) children^5,21^. However, in the study by Wei et al.^22^, concerning 9 hospitalized infants, seven patients were female. Nevertheless, this paper is limited by the small size of the study group.

Infants are a very special group of patients due to close household contacts and the possibility of infection from other relatives, including children or adults^27,29^. It has also been demonstrated that high levels of the SARS-CoV-2 virus have been observed in asymptomatically infected children, which may contribute to the transmission of infections. PCR detection of SARS-CoV-2 virus in faeces has shown that viremia persists for more than 30 days in an asymptomatic child with a negative PCR test from the respiratory tract^30^.

In the SARSTer-PED study, 59% of the infants had documented contact with another infected person in their household, which confirms, as with other researchers, the importance of virus transmission among household members. Feng et al.^31^ published results confirming transmission of infection in households in 56% of children with COVID-19.

In our study, 57% of the infants were diagnosed simultaneously with a family member, which may indicate that infants are as susceptible to infection as adults and that they developed infection at a similar time as adults in the household. This is confirmed by the study by Laws et al.^28^, in which a similar transmission time of infections was observed in children and adults.

In the SARSTerPED study, in 12% of the children with COVID-19, infections in other household members were confirmed after the diagnosis in the children. Therefore, it can be assumed that the incubation time in the infants was shorter, or that the infants were the source of infection for the rest of the household. Similar conclusions about the possibility of infection of parents from their 3-month-old child were published by Cai et al.^32^.

Yonker et al. published the results of research on the high SARS-CoV-2 viremia in infants and, at the same time, low expression of the ACE2 receptor, which results in a low indicator of infection rate in this age group. Their research showed high concentrations of the SARS-CoV-2 virus in the upper respiratory tract of children during the first two days of the acute phase of infection, asymptomatic or with mild symptoms. They concluded that children could be a potential source of infection despite mild disease symptoms^33^. Research by Laws et al.^28^ on the transmission patterns of infections in households indicated the possibility of transmission from children to adults at 20%, and from child to child at 17%. In addition, Mannheim et al.^34^, studying the routes of infection in households among patients in Chicago, Illinois, estimated the possibility of transmission both from child to adult and between children at 13%.

Most infected children have a good prognosis, and symptoms resolve within 1–2 weeks, but severe COVID-19 cases have also been reported^22,35–37^. This is also confirmed by our observations.One child was treated in an intensive care unit but did not require mechanical ventilation. 32 (11%) infants had no clinical symptoms, and the diagnoses were made through screening RT-PCR smears.

The epidemiological reports of COVID-19 in the first months of the pandemic mainly concerned the registration of infections in adults and adolescents over 15 years of age. The percentage of confirmed infections in younger children was low^38,39^. Similarly, in Poland, in the study by Raciborski et al.^1^, the lowest infection rate was recorded among the youngest children. However, in a survey by Bialek et al.^5^ on 2572 children with COVID-19 from various areas of the United States in the period from February to April 2020, 15% of those registered were infants. In another cohort study involving over 20,000 American children hospitalized with COVID-19, infants accounted for 20.2% of the patients^16^.

In the SARSTer-PED study, children under 1 year of age made up 23.4% of 1,283 children under 18 with COVID-19. During the first wave, infants accounted for 10.5% (49/465), and during the second wave, 33.9% (251/741). In the study by Yonker et al.^33^, at the time of the first wave, infants constituted 4% of the infected children hospitalized in a Massachusetts hospital. It can be assumed that in the initial phase of the pandemic, infections in infants were few and were milder. However, the authors of a meta-analysis of 24 publications on SARS-CoV-2 infections among Chinese children up to 10 years of age found that they had the same exposure to infection as adults^40^.

At the same time, in the analysis of Lu et al.^41^, concerning 171 children infected with SARS-CoV-2 treated in a hospital in Wuhan, asymptomatic infections were found in 15.8%. Therefore, it should be concluded that the low epidemiological rates of SARS-CoV-2 infections in infants during the first wave may be the result of incomplete identification of the infected as a result of mild and asymptomatic disease courses or lower exposure of infants.

During the second wave in the SARSTer-PED study, a significant increase in illnesses of infants was noted (84% vs. 16%), (*p* = 0.0001), and treatment was associated with a longer hospital stay (*p* < 0.0001), as a result of more severe COVID-19 courses in infants. During the second wave, a lower number of infections was documented because of contacts between infants and infected family members (53% vs. 90%, *p* < 0.0001), which may indicate an increase in viral infectivity at that time. However, it should be remembered that infants do not wear masks and often do not adopt other infection prevention measures, while at the same time, they have close family contacts and may be prone to cross-contamination. In addition, mild and non-specific symptoms do not prompt parents to seek medical attention.

In the SARSTer-PED study, as in other studies, the most common clinical symptom in infants was fever (77%), followed by a cough (40%), and catarrh (37%), but gastrointestinal symptoms were significantly more common than in the cited publications, and affected 31.3% of the children^5,8,17,21,32,41^.

The authors of the studies also emphasize that in most children the symptoms were mild, their duration was shorter than in adults (median 10 days), the patients had a good prognosis, and severe pneumonia occurred sporadically^22,28,35,36^. However, other researchers point out the presence of severe courses of infection in children^42^, and the publication of Feng et al.^26^, on 15 children with COVID-19 (aged 4–14), describes inflammatory lesions in chest computed tomography in patients with mild and asymptomatic infections. In our study, 70 (23.3%) of the hospitalized infants were diagnosed with interstitial pneumonia in the course of COVID-19 based on clinical symptoms and radiological examinations. In 49 (70%) of those infants, the infection was characterized by high fever (> 38.5 °C), in 16 (22.9%) dyspnoea was observed, in 6 (8.6%) patients, oxygen therapy was used (saturation < 94%), and one patient was treated in the ICU, but similar to Kainth et al.^8^, none required mechanical ventilation and no deaths were recorded.

An analysis by Yasuhar et al.^43^ showed that infants with COVID-19 are more likely to develop dyspnoea and pneumonia than other age groups of the pediatric population.

In our study, pneumonia in the course of COVID-19 was more often diagnosed during the second wave (25% vs. 16%). Boys were diagnosed with pneumonia more frequently (*p* = 0.03). 27% of the infants with pneumonia also had gastrointestinal symptoms, and 16% had comorbidities in this group of infants. However, as in other publications, some children were registered with dyspnoea (22.9%) and some required oxygen therapy (8.6%).

In many publications, the authors report gastrointestinal symptoms, such as abdominal pain, vomiting, and diarrhea, which are more common in the pediatric population than in adults, and may be the only symptom of the disease in children^5,44,45^. In China, the first severe case of COVID-19 in a child with acute respiratory distress syndrome began with gastrointestinal symptoms^46^. In a study by Laws et al.^28^, gastrointestinal symptoms occurred in 42% of the patients.

In the SARSTer-PED study, gastrointestinal symptoms due to COVID-19 occurred in 31.3% of the infants. In 18% of the infants, lack of appetite was the reason for hospitalization. It can be assumed that gastrointestinal symptoms are more common in infants than in older children.

In adult patients, comorbidities such as cardiopulmonary disease and diabetes have been defined as risk factors for the severe course of COVID-19^15^. Publications regarding the course of COVID-19 infections in children also confirm that children with concomitant diseases, similarly to adults, are exposed to more severe courses^5,41,47^. In a study of American children, among 345 pediatric cases with information on underlying conditions, 23% had at least one^5^.

In our study, comorbidities occurred in 12% (35/300) of the patients. Similarly, in the study by Laws et al.^28^, 13% of the children had comorbidities, and the most common disease was bronchial asthma. It can be concluded that the low percentage of comorbidities in this group is due to the young age of the patients.

Among the additional diagnoses made in infants with COVID-19 during the first and second waves, the most common were urinary tract infections, viral gastrointestinal infections with confirmed rotavirus, adenoviral and noroviral etiology, and otitis media. An earlier study by Chonmaitree et al.^48^ identified coronavirus etiology of otitis media in children, which is also confirmed by our observations.

Laboratory abnormalities observed in adults, including elevated levels of D-dimers and cytokines (IL-6, IL-10, TNF-alpha), have been associated with severe COVID-19 courses and increased mortality^49^. Henry et al.^50^, in analyses of laboratory abnormalities in children, showed that 69.2% of the children had normal leukocyte counts, while CRP and PCT were increased in 13.6% and 10.6% of the patients, respectively. When analyzing laboratory abnormalities in infants in the SARSTer-PED study, we found elevated CRP > 10 mg/L in 18% (48/268), and PCT > 0.5 ng/mL in 7% (15/216). As in other publications, the most common abnormality was the elevated concentration of D-dimers (in 52%), and IL-6 (in 40%). Yasuhara showed that elevated levels of D-dimers were found more often in infants than in other age groups of children, which may suggest more severe COVID-19 courses in children up to 1 year of age^43^. However, our observations do not confirm these suggestions.

According to the Recommendations of the Polish Pediatric Society and The National Consultant in the field of pediatrics, clinical management of children with mild and moderate COVID-19 courses consists in outpatient care with the recommendation of adequate hydration and antipyretic treatment. Children with more severe disease, with hypoxemia, and children with co-morbidities may require treatment in specialized hospitals^51^.

In the SARSTer-PED study, oxygen therapy was used in 6 (2%) children due to low oxygen saturation (< 94%), and antipyretic drugs were used for 115 (38%) patients, including paracetamol and ibuprofen. Antibiotics were used statistically significantly more often in sick children during the second wave (33% vs. 6%, *p* = 0.0001), which is associated with more severe COVID-19 courses resulting from complications of bacterial superinfection.

## Conclusions

1. COVID-19 in infants usually manifests as a mild gastrointestinal or respiratory infection, but pneumonia is also observed with falls in oxygen saturation, requiring oxygen therapy.
2. Gastrointestinal symptoms are common in infants infected with SARS-CoV-2, and infant appetite disorders may lead to hospitalization.
3. The clinical course of the disease differed significantly between the first and second wave of the pandemic.
4. It seems that infants may play a role in the transmission of SARS-COV-2 infections in households, despite mild or asymptomatic courses, so eating disorders in infants should be an indication for COVID-19 testing.
